# Delivery and Metabolic Fate of Doxorubicin and Betulin Nanoformulations In Vivo: A Metabolomics Approach

**DOI:** 10.3390/metabo15110723

**Published:** 2025-11-05

**Authors:** Mihai Adrian Socaciu, Remus Moldovan, Carmen Socaciu, Flaviu Alexandru Tăbăran, Simona Clichici

**Affiliations:** 1 Department of Radiology and Medical Imaging, Faculty of Medicine, University of Medicine and Pharmacy “Iuliu Hatieganu”, 400347 Cluj-Napoca, Romania; mihai.socaciu@umfcluj.ro; 2 Department of Physiology, Faculty of Medicine, University of Medicine and Pharmacy “Iuliu Hatieganu”, 400347 Cluj-Napoca, Romania; remus_ri@yahoo.com (R.M.); sclichici@umfcluj.ro (S.C.); 3 Faculty of Food Science and Technology, University of Agricultural Sciences and Veterinary Medicine, 400372 Cluj-Napoca, Romania; 4 Department of Biotechnology, Research Centre for Applied Biotechnology in Diagnosis and Molecular Therapy, Biodiatech-Proplanta SRL, 400478 Cluj-Napoca, Romania; 5 Faculty of Veterinary Medicine, University of Agricultural Sciences and Veterinary Medicine, 400372 Cluj-Napoca, Romania; alexandru.tabaran@usamvcluj.ro

**Keywords:** pentacyclic triterpenoids, PEGylated liposomes, Lipid Nanostructured Carriers, diffraction light scattering, ultra high-performance liquid chromatography, Mass Spectrometry, in vivo delivery, sonoporation

## Abstract

**Background**: Betulins (betulin, betulinic acid and lupeol) demonstrated antitumor and chemopreventive activity but showed low bioavailability due to their self-aggregation in hydrophilic environments. To overcome these disadvantages, their incorporation into lipid nanoformulations (PEGylated liposomes and Lipid Nanostructured Carriers (NLCs)) has proven to represent a viable solution. **Objectives:** The purpose of this study is to evaluate the size and incorporation rate of these molecules in nanoformulations, as well as their delivery and metabolic fate (pure betulinic acid versus a standardized extract, TT) relative to Doxorubicin using an in vivo protocol. The investigation extended our previous in vitro investigations towards an in vivo evaluation of antitumor activity, metabolic fate and toxicity in Wistar rats bearing Walker 256 carcinoma tumors over 21 days. Since previous studies used oral or intratumor administration, this exploratory study applied intravenous administration via microbubble-assisted sonoporation, considering its higher relevance for translational studies. **Methods:** The delivery and metabolic fate of the parent molecules, the identification of their fragments and metabolites using UHPLC-QTOF-ESI^+^MS were investigated, along with the identification of some toxicity biomarkers in rat plasma, tumor tissues and urine. **Results:** Preferential accumulation of Doxorubicin in tumors was observed compared to betulinic acid and TT components, as well as their persistence in plasma or elimination in urine. Compared to PEGylated liposomes, NLC formulations (especially NLC Doxo) induced a lower survival rate, a decreased bioavailability and increased toxicity by around 20%. **Conclusions:** These data are a starting point and complement the contrast-enhanced imaging and histology evaluations, which may contribute to the actual knowledge about the in vivo fate of betulins.

## 1. Introduction

Among pentacyclic triterpenoids (TTs), an important family of bioactive phytochemicals, the Lupane group of molecules (lupeol, betulin and betulinic acid), hereafter referred to as “Betulins”, is synthesized by some plant species and mainly includes betulinic acid and betulin. These molecules proved to have various biological functions, including antioxidant, anti-inflammatory, antiviral, anticancer and hypoglycemic activities, being known as potent pharmacological agents [1,2,3,4,5,6]. The outer bark of *Betula Pendula Roth* (silver birch tree) is one of the richest sources of betulin (B), which constitutes up to 25% of the bark, along with lupeol (LU) (up to 1.5%) and betulinic acid (AB). Their identification, isolation, bioavailability, self-assembly behavior and applications are well documented [1,7,8,9].

The major disadvantage of pentacyclic triterpenes is their low solubility and, therefore, low bioavailability. However, their ability to form self-assembled 3D nanosized structures, enabling them to be used as delivery vehicles, is limited. Some were recognized by the US Food and Drug Administration as Generally Recognized as Safe (GRAS) for oral or topical formulations with low side effects [9,10,11]. There is no single, comprehensive “GRAS list” specifically for all edible pentacyclic triterpenes, but many individual triterpenes from food sources are considered GRAS due to a long history of use. Betulin is not currently listed by the FDA for food applications, but it is considered a safe food additive in many countries, including the U.S., when used within specific dosage limits. It is a natural compound found in birch tree bark and is used as a nutritional supplement.

Scientific interest in AB and B as chemopreventive and antitumor agents has significantly increased after confirming their effects against human melanoma in vitro and in vivo [4,12,13,14]. AB is the most studied molecule with a prominent efficiency in cancer therapy, explained by inhibition of topoisomerases, changes in mitochondrial membrane potential, induction of apoptosis, inhibition of angiogenesis and DNA polymerase, activation of caspases and production of reactive oxygen species [13].

In recent decades, nanoparticle-based drug delivery has been extensively exploited for cancer treatment, mainly via oral or topical administration, due to its improved stability and biocompatibility, enhanced permeability and retention effect and precise targeting [15]. Multiple lipid nanodelivery formulations include liposomes, Solid Lipid Nanoparticles (SLNs), Lipid Nanostructured Carriers (NLCs) and nanoemulsions. Updated information about new nanoformulations for terpenoid delivery in anticancer therapies has been recently published [1,10,11,14,15,16,17].

Some liposomal systems incorporating AB have induced cytotoxicity and apoptosis, disrupted mitochondrial membrane potential in vitro and inhibited tumors in vivo in mice following intratumoral injection [4,18,19,20]. Better stability has been noticed in Polyethylene Glycol-modified (PEGylated) stealth liposomes, which improved AB or B availability in vitro [18,21]. Also, NLCs, which include mixtures of physiological lipids, have shown enhanced targeting as nano delivery formulations [22,23,24] due to their biocompatibility and low toxicity [25,26,27].

Doxorubicin (Doxo) is a well-known anthracycline chemotherapy drug considered the “gold standard” for treating various carcinomas. However, its use as a hydro-chlorinated formula is limited by significant side effects like cardiotoxicity and myelosuppression [2,11,28]. PEGylated Liposomal Doxo has proven higher efficiency and reduced cardiotoxicity compared to free Doxo, along with improved molecular targeting and tumor recognition [29,30,31,32]. Metabolites of Doxorubicin, e.g., doxorubicinol (Doxol) and doxorubicinone (Doxone), have been identified in plasma and tissues using Liquid Chromatography–Tandem Mass Spectrometry in mouse plasma after administration of PLGA–Doxo and compared with a commercial Doxo solution and a liposomal Doxo formulation [33,34,35,36].

Metabolomics is an emerging technology (based on high-throughput techniques like GC-MS, LC-MS or NMR) and an advanced tool for profiling a specific biofluid or targeting specific metabolites and putative biomarkers after in vitro or in vivo treatments. To evaluate the composition and stability of AB and B in organic solvents or in different nanoformulations, several analytical methods were applied, including Gas or Liquid Chromatography coupled with UV–VIS detection or Mass Spectrometry (HPLC–DAD or UHPLC–MS), Fourier Transform Infrared (FTIR) spectroscopy, Near-Infrared spectroscopy (NIRS) and Raman Spectroscopy [13,21,37,38].

The diagnostics and therapeutics for different drug-delivery systems with high potential to identify and treat tumors in vivo often apply CEUS (Contrast-Enhanced Ultrasonography) to visualize their effects, using Microbubbles (MBs) as contrast agents (2 to 10 µm) and vehicles to enhance drug delivery, by sonoporation [39,40,41]. The co-incubation of nanoparticles with MB and especially the conjugates of nanoparticles with MBs showed enhanced cellular uptake of nanoparticles, dependent on many parameters [42,43,44,45].

Our group has previous experience in preparing, characterizing and standardizing birch extracts (TT) enriched in betulin, betulinic acid and lupeol [1,7] as well in building PEGylated liposomes and NLC, which incorporated these molecules and showed acceptable cell delivery and apoptosis in vitro on melanoma and Walker 246 tumor cells [18,21,25,46]. In this study, the size of new nanoformulations with entrapped Doxo, AB and TT was determined by diffraction light scattering and entrapment efficiency, as well as by UV spectrometry and UHPLC-QTOF-ESI^+^MS. Considering the knowledge gaps related to their effects in vivo by parenteral administration of nanoformulations containing Doxo, AB and TT, this study focused on a metabolomics exploratory approach looking for the fate of these drugs as such or their metabolites in vivo, on Wistar rats bearing Walker 256 tumors. Intravenous administration was achieved with different liposomal and NLC nanoformulations co-incubated with microbubbles for a delivery assisted by sonoporation. Using UHPLC-QTOF-ESI^+^MS technology, the presence and fate of AB, TT, Doxo and their metabolites were identified in tumor tissues, plasma, or eliminated in urine. We also identified some metabolic biomarkers (creatinine, p-cresyl sulfate and creatine, arginine and dimethylarginine, hippuric acid and indoxyl sulfate) in order to evaluate comparatively the toxicity of all nanoformulations. These data will correlate with ultrasonography and histology results, which will be reported in a future paper.

## 2. Materials and Methods

### 2.1. Chemicals and Reagents

Betulin (B) and Betulinic acid (AB) (99% purity) were purchased from Roth GmbH Germany. Lupeol (LU) (99% purity) was purchased from Merck GmbH, Darmstadt Germany. Doxorubicin hydrochloride (Doxo) 2 mg/mL saline solution was purchased as a commercial perfusion solution (Accord Healthcare Ltd., Uxbridge, UK). HPLC-grade solvents Methanol, Ethanol, iso-Propanol, Acetonitrile, Formic acid, Stearic acid, Oleic acid, Triethanolamine Dimethyl sulfoxide (DMSO) were purchased from Merck, Darmstadt, Germany. PEG 2000, Triton X-100, Tween80, Lecithin (>98%), and cholesterol were purchased from Sigma Aldrich, Darmstadt, Germany. Compritol 888ATO was provided by Gattefosse, Saint-Priest, France.

### 2.2. Preparation and Composition of Enriched Birch Bark Extract (TT)

The bark of silver birch was collected by the authors of the study from a forest in the Apuseni region (Transylvania, Romania). The dried outer layer (5 g) was grounded, washed 2× with petroleum ether to eliminate resins and the terpenoids were extracted successively with 2 × 100 mL mixture of iso-propanol: ethyl acetate, 1:1 (*v*/*v*), 48 h at 50 °C. After evaporation, the residue was re-extracted in a solvent mix of ethanol:DMSO (3:1). This stock extract (TT) was used in further experiments. Its preliminary evaluation was carried out by UV spectrometry (range 200–320 nm) as compared to pure standards AB and B, calibrations being performed at four different concentrations (1 to 10 mg/mL) either by UV spectrometry or by HPLC-DAD [21]. The total concentration of terpenoids in TT was calculated from maxima absorption at 212 nm and the value obtained was 28.35 mg/mL. Meanwhile, the mean value from a triplicate measurement by HPLC-DAD indicated a concentration of 25.4 mg/mL, with a ratio of B:AB:Lupeol of 55:35:10 (w:w:w). This value was considered for further experiments.

### 2.3. Preparation of PEGylated Liposomes Using the Ethanol Injection Method

Details about the procedure applied by Ethanol injection method were reported previously [18]. Empty control liposomes and liposomes containing TT (L-TT) and AB (L-AB) were prepared, using 2 mL AB (50 mg) and 2 mL TT (50.8 mg) dissolved in the same solvent mix and introduced in lipid phase, just before being added to the aqueous phase. A volume of 35.5 mL liposomal suspension was obtained, the theoretical concentration of AB and TT in liposomes being 1.40 mg AB/mL and 1.43 mg TT/mL, respectively. To obtain L-Doxo a volume of 5 mL Doxo (10 mg) was dissolved in 35 mL aqueous phase and then 16 mL lipid phase was added, following the same procedure. In this case, a volume of 40 mL liposomal suspension was obtained with a theoretical concentration of 0.25 mg/mL.

### 2.4. Preparation of NLC Formulations by Melt Emulsification

To obtain the NLC formulations, the melt emulsification procedure was applied, as described previously [18,36]. The resulting suspension was kept on ice for 15 min. To obtain NLC-AB and NLC-TT formulations, 28 mg AB and 1 mL TT (25.4 mg) were dissolved in ethanol: DMSO (3:1), introduced in 20 mL hot water, and added dropwise to the melted lipid mix. Similarly, NLC-Doxo was obtained using 40 mg saline Doxo diluted in water. The final NLC suspensions had a volume of 20 mL. The theoretical concentrations were 1.4 mg AB/mL NLC suspension, 1.3 mg TT/mL and 2 mg Doxo/mL.

### 2.5. Experimental Design and Steps for the Characterization of Nanoformulations and Co-Incubation with Microbubbles

**Figure 1:**
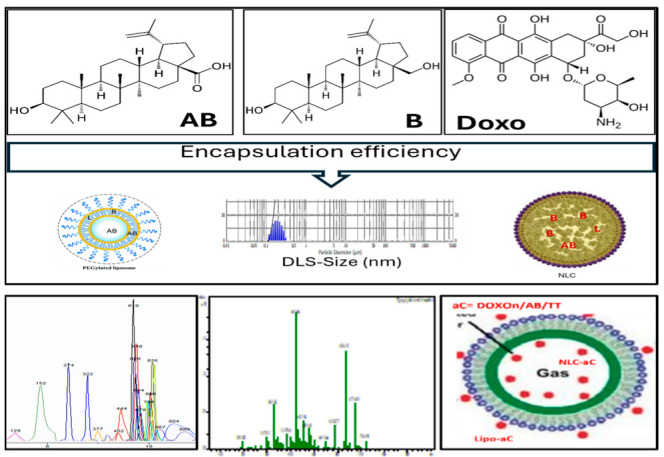
Main steps applied for the characterization of nanoformulations (liposomes and NLC) with encapsulated AB, TT extract and Doxorubicin: size DLS measurement, encapsulation efficiency (EE%), followed by UHPLC-QTOF-ESI^+^MS analysis, before and after co-incubation with MBs.

Figure 1 summarizes the main steps followed for the characterization of nanoformulations (liposomes and NLCs) which incorporated the TT extract (mix of B, AB and L) and comparatively pure standards of AB and Doxo.

### 2.6. Entrapment Efficiency and Size Determination of PEGylated Liposomes and NLCs

To evaluate the entrapment efficiency (EE%) of Doxo, pure AB and TT inside each formulation (L- and NLC-), 10 mL from each L- or NLC- suspension was filtered under centrifugation (Hettich Rotofix 46 Centrifuge, 4600 rpm, 30 min at 25 °C) through Amicon^®^ Ultra 15 mL Centrifugal Filter devices (Millipore, Burlington, VT, USA) with 100 K cut-off. The retentate was collected after two successive washings. After restoration of the initial volume of 10 mL, the absorption values (recorded at 212 nm by UV-VIS spectrometry) of AB and TT in the retentate were determined after dissolving in a solution of 0.1% Triton X-100 in ethanol and compared to the absorptions of the initial suspensions dissolved in the same solvents. The entrapment efficiency was calculated by the EE% = (A_i_ − A_free_/A_i_) × 100, where A_i_—absorbance of initial suspension; A_free_—absorbance of non-encapsulated fraction. The sizes of all nanoformulations and the Polydispersibility Index (PDI) were also determined by laser diffraction technique using the Shimadzu SALD 2300 DLS instrument (Shimadzu, Kyoto, Japan) and the software Wing SALDII version 3.4.10., as previously described [18,21]. Table 1 includes this data.

### 2.7. UHPLC-QTOF-ESI^+^MS Analysis

This analysis was performed either for free molecules (pure standards of AB, B, Doxo) or their nanoformulations (variant 1), as well as for their identification in plasma, urine and tumor extracts (variant 2). In both cases we used the Bruker Daltonics MaXis Impact (Bruker GmbH, Bremen, Germany) device including a 7.2. (Waltham, MA, USA) with a quaternary pump delivery and QTOF-ESI^+^MS detection.

For variant 1, a C_18_ reverse-phase column (Kinetex, UPLC, Torrance, CA, USA) (5 µm, 4.6 × 150 mm) at 25 °C was used, with a flow rate of 0.8 mL/min and an injection volume of 25 µL. The mobile phase was represented by a gradient of eluent A (pure water containing 0.1% formic acid) and eluent B (Methanol:Acetonitrile:Isopropanol, 1:1:1, containing 0.1% formic acid). The gradient system consisted of 70% A (min 0), 30% A (min 4), 0% A (min 7), 30% A (min 10), 70% A (min 13), followed by 2 min isocratic elution with 70%A. The total running time was 15 min.

For variant 2, the precursor molecules and their metabolites were separated on an Acclaim C_18_ column (5 μm, 2.1 × 100 mm, pore size of 30 nm) (Thermo Scientific, Waltham, MA, USA). The mobile phase consisted of 0.1% formic acid in water (A) and 0.1% formic acid in acetonitrile (B). The elution time was set for 20 min. The flow rate was set at 0.3 mL·min^−1^. The gradient for serum samples was 90 to 85% A (0–3 min), 85–50% A (3–6 min), 50–30% (6–8 min), 30–5% (8–12 min) and increased to 90% at min 20. The volume of injected extract was 5 µL, and the column temperature was 25 °C. In parallel QC samples were analyzed for reproducibility. All measurements were carried out in duplicate.

In both variants, the MS parameters were set for a mass range of 100–600 Da. The MS parameters were set on ionization mode positive (ESI^+^), capillary voltage 3500 V, pressure for the nebulizing gas 2.8 Barr, drying gas flow 12 L/min, and drying gas temperature 300 °C. The control of the instrument and data processing used the specific software provided by Bruker Daltonics, namely Chromeleon 7.2, TofControl 3.2, Hystar 3.2 and Data Analysis 4.2. The identifications were performed by Data Analyses 4.2, considering the MS spectral analysis for each component and specific fragmentation. The Extract Ion Chromatogram was also used to confirm the specific fragments for B, AB, Doxo and their metabolites in nanoformulations as well in plasma, urine and tumor extracts.

### 2.8. Statistical Analysis

By UHPLC-QTOF-ESI^+^-MS analysis, we separated around 800 molecules in plasma and around 750 in urine and tumor extract. By Data Analysis, the TIC+MSn (Total Ion Chromatograms) were obtained and all acquired data were processed. Using the algorithms Find Molecular Features (FMF) and Extracted Ion chromatograms (EIC), the [M+1] values, the retention time, the peak area, the peak intensity and the signal/noise (S/N) ratio were determined for each molecule. The statistical analysis was based on FMF matrices. In the first step, the MS peaks having retention times under 1 min and more than 15 min were eliminated, and intensities below 3000 units, S/N values < 10, and *m*/*z* values above 600 Daltons were removed. In the second step, the alignment of *m*/*z* values was performed by the online software https://www.bioinformatics.org/bioinfo-af-cnr/NEAPOLIS/ (accessed on 30 August 2024), keeping the common molecules found in more than 75% of the samples. After using the procedures mentioned above, *m*/*z* values were targeted corresponding either to possible metabolites of AB, B, Doxo in all samples or to different possible toxins and from these matrices the ANOVA analysis on the Metaboanalyst 6.0 platform was carried out, to identify the statistically significant differences between samples and groups.

The identification of molecules and fragmentation was confirmed by International Databases such as Human Metabolome Database (https://www.hmdb.ca/ (accessed on 2 October 2024)), Lipid Maps (http://www.lipidmaps.org (accessed on 3 October 2024)). The accuracy of *m*/*z* values (theoretical–experimental) was below 20 ppm.

The representation of statistical relevance was carried out by Principal Components Analysis (PCA), Partial Least Squares Discriminant Analysis (PLSDA), Random Forest (RF)-based prediction test and illustrated by Heatmap clusters and correlations. The values of Variable Importance in the Projection (VIP) and graphs of RF-Mean decrease accuracy (MDA) scores were calculated and ranked for top 15 molecules. ANOVA analysis was applied in the untargeted metabolomic context and the Fisher LSD post hoc analysis (*p*-value cutoff of 0.05) for the most relevant metabolites.

### 2.9. Animals and Experimental Design for In Vivo Delivery

The experiment was carried out on 54 healthy, adult, male Wistar rats, weighing 330 ± 20 g. Animals were obtained from the Biobase of “Iuliu Haṭieganu” University of Medicine and Pharmacy Cluj-Napoca, Romania and then they were housed in an appropriate environment in the Vivarium of the Physiology Department, Faculty of Medicine: a constant temperature of 25 °C, 35% humidity and 12 h light/dark cycles with a standard diet and free access to water. The in vivo experiments were performed in the Imaging laboratory of the Research Center for Applied Biotechnology and Molecular Therapy Proplanta Ltd. (www.biodiatech.ro (accessed on 5 November 2024). All experiments were approved by the Ethics Committee of “Iuliu Hatieganu” University of Medicine and Pharmacy Cluj-Napoca (approval nr.375/6.07.2023) and performed in accordance with ARRIVE guidelines, with the European Directive 2010/63/UE regarding the protection of the animals used for scientific purposes, with the National Veterinary legislation and RRR concepts. The ARRIVE check list data and the set of recommendations according to guideline 2.0 for in vivo experiments was respected, for improving the reporting of these studies to maximize reliability.

The rats were randomly divided into 9 study groups (n = 6). We had a control group (Co), and 8 groups inoculated in the thighs with small fragments of Walker 256 breast cell carcinoma tumors under anesthesia. One of the 8 groups was the tumor control group (Ct), without any treatment, and the 7 remaining groups received different treatments, Doxo, LDoxo, NLCDoxo, LAB, NLCAB, LTT, NLCTT, with the dose and injected volume presented in Table 2 for each substance.

After tumors became palpable and visible as subcutaneous masses of 10–20 mm in thickness by ultrasonography (week 4–5 from tumor inoculation), the treatment was performed in 2 administrations (day 1 and day 7), by intravenous treatment under anesthesia. All animals developed the tumor and were included in the study. A general anesthesia was applied in all cases using isoflurane. No drugs for analgesia were used.

Humane end points were set as a decrease in body weight of 20%, an increase in tumor weight at 10% from the body weight and the alteration of clinical condition. Euthanasia was performed by cervical spine dislocation and decapitation. Details according to ARRIVE guideline 2.0 are also presented.

For each group, an initial imaging examination and evaluation (native + CEUS) was performed on day 1 before administration, followed by imaging performed at 1-week intervals (days 7 and 14 after treatment) and the rats were finally examined and then sacrificed after 21 days. Animals were euthanized via cervical dislocation under anesthesia with 90 mg/kg b.w. ketamine and 10 mg/kg b.w. xylazine.

**Figure 2:**
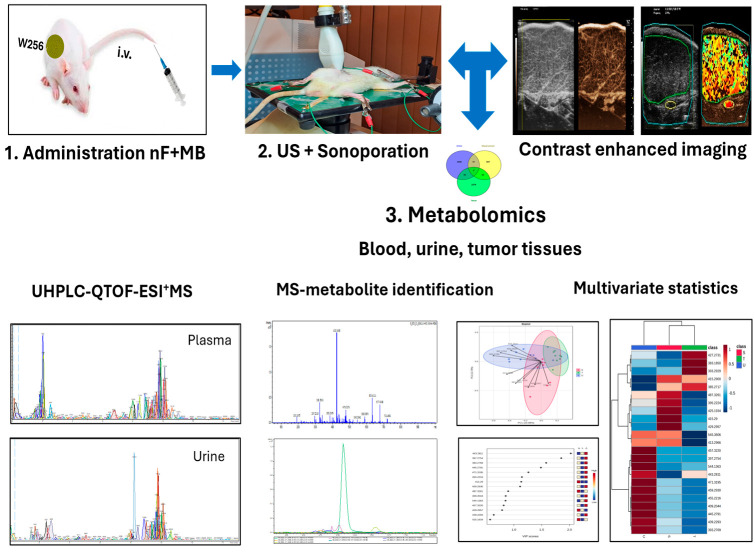
Main steps of the in vivo experiment: intravenous administration of nanoformulations and microbubbles (nF + MB) (1), delivery by sonoporation, examination by CEUS (2), and metabolomics applied by UHPLC-QTOF-ESI^+^MS and multivariate statistics (3).

Figure 2 includes the main steps of the experiment, from intravenous administration of nanoformulations (nFs) co-incubated with microbubbles (MBs) (1), to delivery by sonoporation and Contrast-enhanced imaging (CEUS). For sonoporation we used an ultrasound emitting device used for dermatological purposes (Alveola F-702, Mimera Europe s.r.o, Senek, Slovakia), with a round footprint probe (20 mm diameter), 1 MHZ pulses with 100% duty cycle and fixed mechanical power intensity of 2 W/cm^2^. We chose this approach over an imaging transducer because of the improved efficacy in bubble burst due to higher energy output and much higher duty cycle. The time chosen for sonoporation was 10 min, to allow the replenishment of the tumor territory with fresh microbubble complexes from the blood pool for an efficient delivery.

Finally, the evaluation of parent molecules and metabolites was applied by UHPLC-QTOF-ESI^+^MS technology and multivariate statistics (3). All results obtained by CEUS and histology will be included in a separate manuscript (future paper).

### 2.10. Processing and Metabolomic Analysis of Rat Samples

The blood was collected from the tail vein, and the tumor tissues were collected from each group of rats after euthanasia (day 21), while the urine samples were taken at 12 h after the second administration of samples (day 7). Plasma was obtained by centrifugation for 10 min at 2000× *g* using a refrigerated centrifuge and kept at −20 °C until analysis. Urine was collected using a metabolic cage, monitoring its volume and the water volume consumed by each rat.

For metabolite extraction, a volume of 1 mL mix of pure HPLC-grade Methanol:Isopropanol:Methyl t-butyl Ether (2:1:1) (MPBE) was added for each volume of 0.8 mL of plasma or urine. The mixture was vortexed to precipitate proteins, ultrasonicated for 5 min and stored for 24 h at −20 °C for protein precipitation. The supernatant, collected after centrifugation at 12,500 rpm for 10 min (4 °C), was filtered through Nylon pore of 0.25 μm. Finally, the supernatants were placed in glass micro vials and introduced in the autosampler of the UHPLC before injection. Quality Control (QC) samples from urine or plasma were obtained by mixing 0.2 mL from each sample, run at the beginning and after each 10 samples, for checking the reproducibility of UHPLC-MS analysis. The parent molecules and their fragments were identified with pure standards, as presented in the Supplementary File.

Aliquots of approx. 1 g Walker 256 tumor tissue were mixed with 2 mL PBS buffer (pH 7.2) and vortexed vigorously, then placed in an ultrasonic bath at 37 °C for 30 min, kept at 4 °C for 6 h, and centrifuged at 12,500 rpm. The supernatant was separated and stored at −20 °C until analysis. The pellet was treated with 1 mL of 2.5% trypsin in PBS buffer, stored at 37 °C for 4 h, and then kept at −20 °C for 12 h. Finally, the supernatant and the trypsin-treated suspension were mixed, vortexed and extracted in 5 mL MPBE solvent.

Calibrations were made in parallel with stock solutions of AB (3 mg/mL MPBE), B (7 mg/mL MPBE) and Doxo (2 mg/mL MPBE), respectively. Parent ions, fragments and metabolites of AB, B and Doxo were identified and evaluated.

## 3. Results

### 3.1. Characterization of Nanoformulations

Table 1 includes data about the size distribution (mean diameter ± SD; nm) of liposomal (L) and NLC formulations, the poly dispersibility index (PDI) and the entrapment efficiency (EE%) of Doxo, AB and TT. The final concentrations (Cf) were mentioned in each case. The size of microbubbles (MBs) used were also measured.

**Table 1:** The size distribution and diameters (mean ± SD; nm), PDI, EE% and final concentrations (Cf) for Doxo, AB and TT in liposomal (L) and NLC formulations used in this study. For details see Materials and Methods.

** 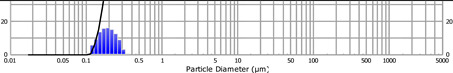 **	L-Control: 192 ± 20 nm PDI = 0.104
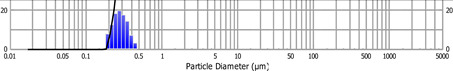	LDoxo: 288 ± 25 nm PDI = 0.087 EE% = 87.5%; Cf = 1.75 mg/mL
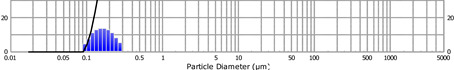	LAB: 177 ± 25 nm PDI = 0.140 EE% = 71.4%; Cf = 1 mg/mL
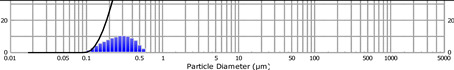	LTT: 198 ± 43 nm PDI = 0.207 EE% = 70%; Cf = 1 mg/mL
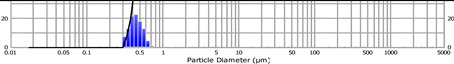	NLC-Control: 452 ± 18 nm PDI = 0.040
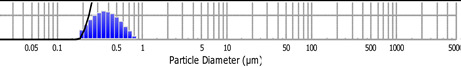	NLCDoxo: 385 ± 54 nm PDI = 0.140 EE% = 50%; Cf = 1 mg/mL
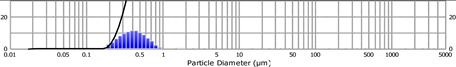	NLCAB: 403 ± 52 nm PDI = 0.124 EE% = 65%; Cf = 0.9 mg/mL
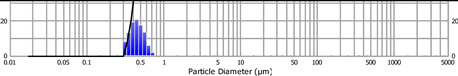	NLCTT: 455 ± 22 nm PDI = 0.050 EE% = 70.8%; Cf = 0.9 mg/mL
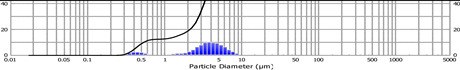	MB: 3500 ± 1400 nm PDI = 0.160

The mean sizes of nanoformulations ranged from 177 to 455 nm, depending on the type of nanostructure and the terpenoid encapsulated. The PDI showed a good distribution of nanoparticles in all cases and ranged from 0.05 to 0.207, being higher in NLC formulations as compared to L-formulations. The smallest L-structures entrapped AB, TT and Doxo with higher EE% (70 to 87.5%), while NLC formulation had larger sizes and lower EE% (50 to 70.8%), being cloudier with increased tendency for aggregation as compared to Liposomes. NLCAB had a mean diameter of 403 nm and EE% = 65%, while entrapped TT showed mean sizes of 455 nm and EE% = 70.8%. The TT extract, being less polar than AB, due to its higher content of betulin and lupeol, showed a better encapsulation into NLC matrix as compared to Doxo and AB. The stability of both types of nanoformulations was high, with constant sizes, as checked for one month storage at 4 °C. Significant correlations were found between microscopic evaluation and the DLS measurements, as reported previously [18].

### 3.2. Evaluation of Rat Survival, Tumor and Body Weight, Water Consumption

Table 2 includes data about the samples (code, volume and dose injected) for each group of 6 rats, the survival rate, rat weight before euthanasia, tumor weight. The water consumption and the volume of urine were also monitored.

**Table 2:** The samples (code, volume injected and dose), the survival rate, mean rat weight before euthanasia, mean tumor weight. The water consumption and the volume of urine were also monitored. C_0_—healthy controls; C_t_—tumor bearing rats.

Sample Codes	Injected (mL)	Dose (mg/kg)	Survival (%)	Mean Weight (g)	Tumor Weight (g)	Water Consumed (mL)	Urine Volume (mL)
C_0_	-	0	100	304.5 ± 2.6	-	35	12.0
C_t_	-	0	100	365 ± 40.1	8.5 ± 4.28	40	12.5
Doxo (2 mg/mL)	1	6	100	344 ± 30.0	6.0 ± 1.41	-	-
LDoxo (1.75 mgDoxo mL)	1.2	6	100	372 ± 42.1	8.0 ± 2.92	30 (12 h)	10 (12 h)
NLCDoxo (1 mg Doxo/mL)	2	6	40	335 ± 35.1	11.3 ± 1.71	32 (12 h)	10.5 (12 h)
LAB (1 mg AB/mL)	2	6	75	359.6 ± 6.4	23.3 ± 10.5	<10 (12 h)	3 (12 h)
NLCAB (0.9 mg AB/mL)	2	6	60	366.5 ± 7.5	12.2 ± 8.95	28 (12 h)	10.8 (12 h)
LTT (1 mg/mL)	2	6	62.5	370.6 ± 8.6	17.4 ± 8.93	15 (12 h)	5 (12 h)
NLCTT (0.9 mg/mL)	1.5	4	0	-	-	-	-

The rats treated with NLCTT did not survive, and the other groups showed survival rates from 100% (Ct, Doxo, LDoxo) down to 40% (NLC Doxo) after 21 days of surveillance. No significant modifications of rat mean weight after treatment were noticed, excepting the larger SD variation in the case of groups Ct and Doxo-treated groups. The tumor weight ranged from mean values of 6 to 8 g (Ct and Doxo) to 17–23 g after administration of liposomal formulations and less (11–12 g) for NLC formulations. In general, higher weights of tumors were associated with lower water consumption and urine volumes after 12 h. from the last administration (day 7).

### 3.3. Identification of Doxorubicin, AB and TT Fragments and Metabolites

Pure standards of Doxo, Betulin (B), Betulinic acid (AB) and Lupeol (LU) were first analyzed by UHPLC-QTOF-ESI^+^MS, the parent ions [M+1] and fragments being identified (Table 3).

Doxo showed a parent ion of 544.1360 Da, but also a major fragment of 397.0619 Da (Doxof1). Betulin (B) was identified by its parent ion with 443.3068 Da and the dominant fragment of 425.3474 Da (100%) (Bf1), in addition to a fragment of 393.2769 Da (Bf2), while Betulinic acid (AB) parent ion had 457.6238 Da (100%) and fragments of 439.2293 (ABf1) and 429.2957 (ABf2). Lupeol (LU) had a parent ion of 427.27313 Da and two specific fragments at 411.2787 Da (LUf1) and 383.2850 Da (LUf2).

The Retention times (Rt) and peak intensities were recorded and expressed per ml of plasma or collected urine, as well as per g of tissue. To confirm the metabolites identified in these samples, the [M+1] values > 380 Da were compared with data obtained from other investigations on pure betulin and betulinic acid, as reported previously [37,38]. The parent molecules, their fragments and metabolites were identified and are presented in Table 4.

The Supplementary File S1 includes the EIC chromatograms derived from UHPLC-QTOF-ESI^+^MS analysis for Doxo, AB, TT metabolites identified in plasma (P), urine (U) and tumor (T) tissues. The MS analysis may distinguish between molecules’ fragments (resulting during ionization of the parent molecules) presented in Table 3 and metabolites (intact molecules resulting from the breakdown of parent molecules by different biochemical reactions).

### 3.4. ANOVA Statistics Applied for Doxorubicin, AB and TT Nanoformulations

**Figure 3:**
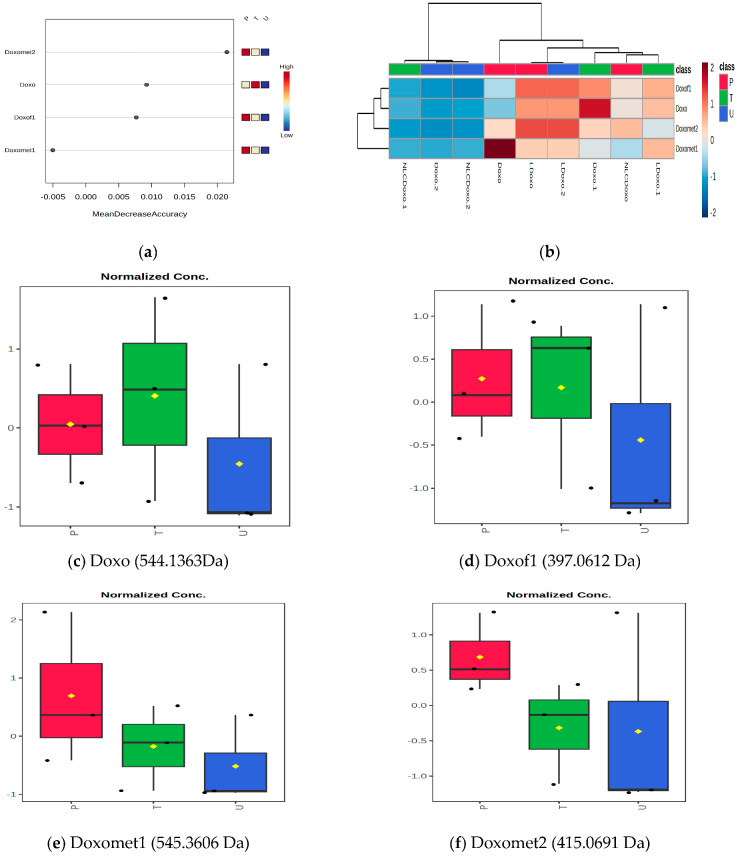
(**a**) The mean decrease accuracy scores in the RF plot; (**b**) the heatmap including clusters (upper side) and relative levels of Doxo and metabolites in each sample. Doxo, LDoxo and NLCDoxo represent Doxo levels in Plasma; Doxo.1, LDoxo.1 and NLCDoxo.1 represent Doxo levels in Tissue; Doxo.2, LDoxo.2 and NLCDoxo.2 represent Doxo levels in Urine; (**c**–**f**) Normalized ANOVA Fisher LSD graphs for mean levels of parent Doxo (**c**), its Doxof1 (**d**) and the two metabolites Doxomet1 (**e**) and Doxomet2 (**f**) in P, T and U.

Based on the UHPLC-QTOF-ESI^+^MS data, the metabolomic ANOVA Analysis of Variance was applied, using the Metaboanalyst 6.0 platform. Figure 3 includes data about the Doxo, as parent molecule, its main fragment Doxof1 and metabolites Doxomet1 and Doxomet2 in plasma (P), urine (U) and tumor tissue (T) after the i.v. administration with Doxo, as a free molecule as compared to LDoxo and NLCDoxo. Figure 3a includes the RF plot, reflecting the mean decrease accuracy scores, while Figure 3b shows the heatmap illustrating the clusters (upper side) and the relative levels of Doxo fragments and metabolites in each sample (P, T or U). Figure 3c–f show the normalized graphs obtained by ANOVA post hoc analysis (Fisher’s LSD; *p*-value cutoff of 0.05) for parent Doxo, its main fragment (Doxof1) and the two metabolites Doxomet1 (Doxorubicinol, 545.1362 Da) and Doxomet2 (Doxorubicinone, 415.0691 Da).

According to Figure 3a, in plasma higher levels of Doxo and its metabolites were identified, followed by tissue and less in urine. The heatmap shows, when compared the administration of free Doxo with LDoxo and NLCDoxo, that free Doxo was prevalent in plasma and tissue, while the Doxo released from liposomes was mainly identified as Doxomet2. The lowest levels of Doxo were identified when NLCDoxo was injected, suggesting its lower bioavailability. The ANOVA post hoc Fisher’s LSD analysis confirmed that Doxo was stored best in tissue (Figure 3c,d), while Doxo metabolites (Doxomet1 and Doxomet2) were found mainly in plasma (Figure 3e,f). In both cases, the highest accumulation was noticed when Free Doxo and LDoxo were injected, without release in urine.

**Figure 4:**
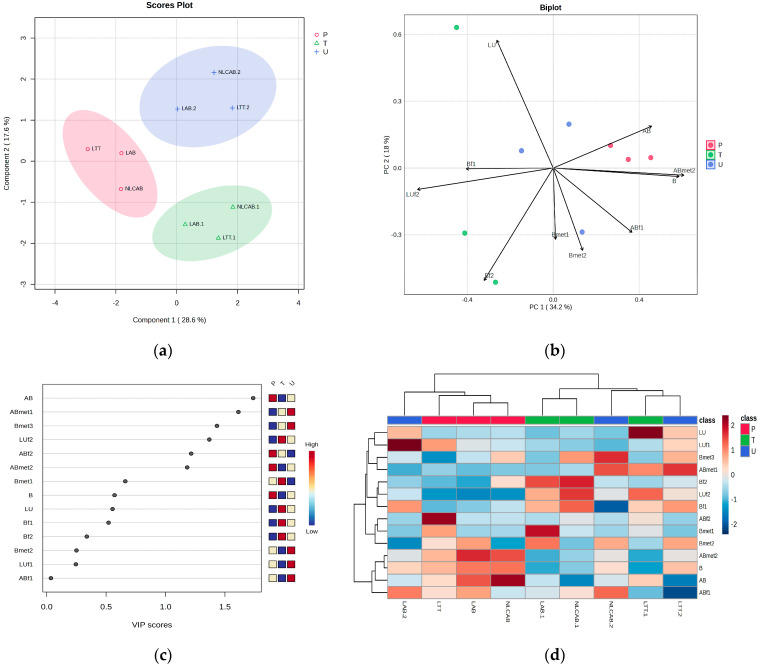

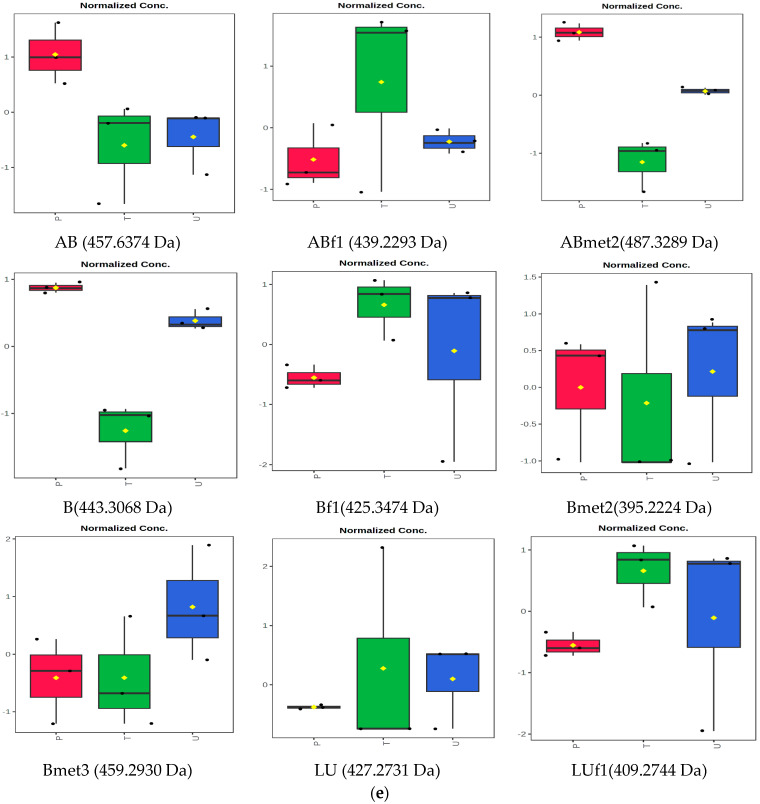
ANOVA statistics to compare the levels of AB and TT (B + AB + LU) fragments and metabolites in P, T and U. (**a**) PLSDA score plot; (**b**) PCA biplot; (**c**) VIP scores; (**d**) heatmap. LAB, NLCAB and LTT represent AB and TT levels in Plasma; LAB.1, NLCAB.1 and LTT.1 represent AB and TT levels in Tissue; LAB.2, NLCAB.2 and LTT.2 represent AB and TT levels in Urine. (**e**) Normalized Fisher LSD graphs for mean levels of parent AB, B, LU and their fractions and metabolites with corresponding [M+1] values in parentheses.

A similar ANOVA Analysis was applied to compare the levels of AB and TT (B + AB + LU) in P, T and U. Figure 4 includes the PLSDA score plot (a), the PCA biplot (b), VIP scores (c), the heatmap (d). The normalized graphs obtained by ANOVA post hoc analysis includes the mean levels of parent AB, B, LU, their fractions and metabolites in P, T and U. For details about fragments and metabolites, see Table 4.

According to Figure 4a, significant discrimination between the sample profiles was noticed, with a co-variance of 46.2%. The discrimination was explained by the biplot and VIP scores (Figure 4b,c), which shows that the molecules explaining the discrimination, namely in plasma, were dominantly found to be parent molecules of AB and B (from LTT) and ABmet2 (originating from LAB and LTT). In urine, more polar metabolites (ABmet1, Bmet2 and Bmet3) were mostly eliminated and in tumor tissue less polar ones originating from LTT were accumulated (LUf2, Bf1, Bf2). The heatmap shows the distribution of these molecules in individual samples and shows a homogeneous plasma cluster for LAB, NLCAB and LTT. Complementary information is offered by ANOVA post hoc graphs (Figure 4e) where the statistical variation of relevant molecules identified in P, T and U was obtained. The levels of AB and one of its metabolites (ABmet2) as well as B (originating from LTT) were dominant in plasma, while B and its metabolites were mostly eliminated in urine. The parent molecules of B, LU originating from LTT and AB delivered from NLC formulation showed lower accumulation in tumor tissue, suggesting their low availability.

### 3.5. Distribution and Semi-Quatitative Evaluation of the Antitumor Molecules and Their Metabolites in Plasma, Tumor Tissue and Elimination in Urine

**Figure 5:**
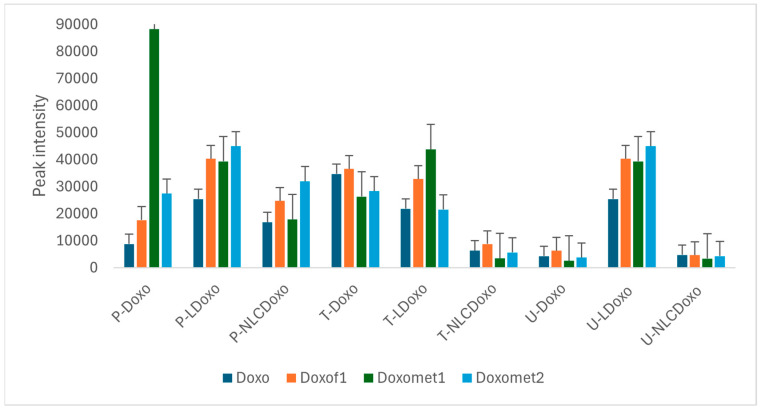
Distribution of Doxo parent molecule, Doxof1, Doxomet1, Doxomet2 (expressed in LC-MS peak intensity) in plasma (P), tumor tissue (T) and urine (U), after the i.v. administration of free Doxo as compared to its delivery from Liposomes (LDoxo) and NLC (NLCDoxo).

**Figure 6:**
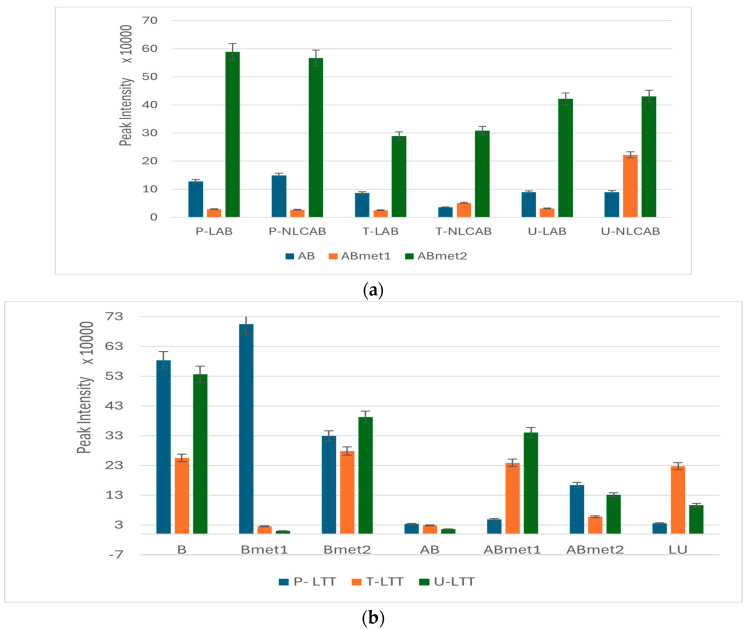
Distribution (expressed in LC-MS peak intensity) of parent molecule AB and their metabolites in P, T and U, after LAB and NLCAB administration (**a**) and a similar distribution of B, AB and LU and their metabolites in P, T and U, after LTT administration (**b**). For abbreviations see Table 4 and Section 2.

To obtain a better explanation of the metabolization and distribution of these molecules, based on UHPLC-MS peak intensities, a semi-quantitative evaluation of the levels of antitumor molecules (Doxo, AB, B, LU) and their metabolites in plasma (P), tumor (T) or elimination in urine (U) was achieved. The distribution of Doxo (parent + Doxof1), Doxomet1 and Doxomet2 is presented in Figure 5 and the distribution of AB, TT components (B, AB and LU) and their metabolites is presented in Figure 6.

As suggested by the data presented, not only the intact Doxo molecule, but also its metabolites (Doxomet1 and Doxomet2) may play an important role in the accumulation in Walker 256 tumor, plasma and release in urine. When free Doxo molecule was administrated, the accumulation of Doxo and its metabolites was almost equally distributed in T (T-Doxo), while Doxomet1 was dominant in plasma (P-Doxo). Very low Doxo levels were noticed in urine. When LDoxo was administrated, the accumulation of Doxo and its both metabolites were almost equally distributed in T and P, and its polar metabolites Doxomet1 and Doxomet2 were largely released in urine (U-LDoxo). The administration of NLCDoxo induced a low retention in tumor and release in urine, but superior distribution in Plasma, its bioavailability being lower than for free Doxo or LDoxo. This finding was correlated with a lower survival rate of the NLCDoxo group of rats and the toxicological data.

Figure 6a illustrates the distribution (expressed in LC-MS peak intensity) of parent molecule AB and their metabolites in P, T and U, after LAB and NLCAB administration. Figure 6b illustrates the distribution of B, AB, LU and their metabolites in P, T and U, after LTT administration.

According to Figure 6a, LAB and NLCAB induced similar behaviors, with a higher retention of ABmet2 in plasma, lower levels in tumor tissue and elimination in urine. ABmet2, the most polar molecule originating from AB proved to be the most available molecule. When LTT was injected, B and Bmet2, ABmet1 and LU showed higher accumulation in tumor tissue. Meanwhile, B and its metabolites (Bmet1 and Bmet2) showed higher retention in plasma. The urine elimination was more important for B, Bmet2 and ABmet1.

### 3.6. Metabolic Biomarkers of Toxicity Identified in Plasma, Tumor and Urine

To evaluate the toxicity induced by the i.v. administration of all nanoformulations, a toxicity investigation was carried out by UHPLC-QTOF-ESI^+^MS untargeted analysis. Applying the protocol variant 2 (see Section 2.7), we identified some molecules as toxicity biomarkers either in plasma or tumor and eliminated in urine. Table 5 includes the list of these molecules, identified by their [M+1] values and confirmed by HMDB ID number.

**Figure 7:**
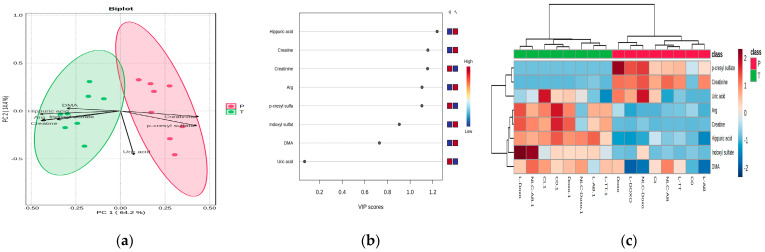
(**a**) PCA biplot; (**b**) VIP scores graph; (**c**) the heatmap including clusters (upper side) and correlations between the levels of the putative toxicity biomarkers in plasma (P) and tumor tissue (T) induced by the formulations used for in vivo administration.

Figure 7 includes the PCA biplot (graphic representation which combines the scores and the loadings obtained by PCA) (a), the VIP scores derived from PLSDA (b) and the heatmap illustrating the correlations between the levels of the biomarkers found in plasma (P) and tumor tissue (T) (c) after the in vivo administration of individual nanoformulations.

With a co-variance of 78.6%, the biplot (Figure 7a) shows significant discrimination between the profiles of plasma (P) and tumor tissue (T), mostly represented by creatinine, p-cresyl sulfate and creatine, Arg and DMA, hippuric acid and indoxyl sulfate, respectively. The VIP scores >1 were represented by creatinine, p-cresyl sulfate (P) versus hippuric acid and creatine (T) (Figure 7b). The heatmap confirms these findings and moreover identifies Doxo, L-Doxo and NLC-Doxo as formulations which increased the levels of creatinine, p-cresyl sulfate and uric acid in plasma. In tumor tissue, the above-mentioned molecules had increased levels when L-Doxo and NLC-AB were injected (Figure 7c).

### 3.7. Distribution and Semi-Quantitative Levels of Toxicity Biomarkers in Plasma, Tumor Tissue and Urine

**Figure 8:**
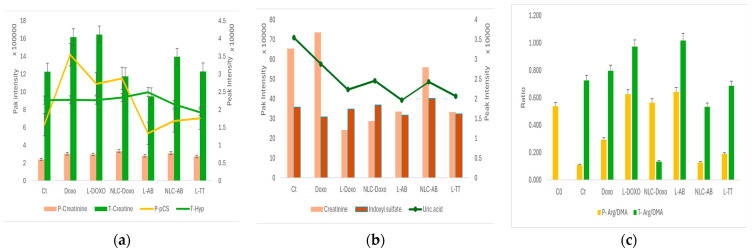
(**a**) Peak Intensity levels of Creatinine and pCS in Plasma (P), Creatine and Hyp in tumor tissue (T). (**b**) Creatinine, Indoxyl Sulfate and uric acid found in urine. The secondary graph axes are represented as lines. (**c**) Ratios of Arg to DMA in P and T.

A semi-quantitative evaluation (expressed as peak intensity) of the statistically relevant biomarkers identified in plasma (P), tumor (T) or elimination in urine (U) is presented in Figure 8.

According to Figure 8a, in plasma the creatinine levels were almost constant, while p-CS was significantly increased as compared to tumor bearing rats (Ct) after administration of Doxo, LDoxo and NLCDoxo. Meanwhile, in tumor tissue creatine levels were increased after administration of Doxo, LDoxo, NLCAB as compared to tumor bearing rats (Ct). Meanwhile, hippuric acid did not show significant variations in tumors.

Figure 8b illustrates the significant increase in creatinine in urine for free Doxo, while liposomal and NLC variants showed significant decreases as compared to the Ct group. Uric acid levels in urine decreased significantly when Liposomal Doxo, AB and TT were administered.

An important criterion of toxicity is considered the ratio of Arg/DMA and this is represented in Figure 8c; as compared to C_0_ (healthy rats), this ratio in plasma (P) significantly decreased for Ct, NLCAB, LTT as an indicator of toxicity. In tumor tissue the ratio did not show significant variations, compared to Ct, excepting NLC Doxo, which indicates a higher toxicity.

## 4. Discussion

The utilization of natural compounds for the prevention and treatment of cancer tumors in vitro or in vivo has attracted significant attention from the scientific community in recent decades. Although the use of nanotechnology in cancer therapy is still in the preliminary stages, the application of improved nanocarriers and nanotherapeutics has been demonstrated to decrease the various limitations related to the use of natural compounds, such as physical/chemical instability, poor aqueous solubility, and low bioavailability. Therefore, nanotechnology has emerged as a promising solution to improve the bioavailability and effects of the natural compounds [47].

The generic name of “betulins” used in this study included three pentacyclic terpenoids from the lupane family, namely betulin, betulinic acid and lupeol, molecules with confirmed antioxidant, antitumor and many other bioactivities. Their low solubility in aqueous media remains a significant limitation, inducing low bioavailability and restricted applications. Chemopreventive and antitumor effects have been observed in several in vitro and in vivo studies (performed mainly in mice, and mostly by oral, topical or intratumor administration), the major mechanism of anticancer action of betulin and betulinic acid being the induction of apoptosis without significant toxicity to normal cells [12,18,41].

Previously, available studies were reported only for Doxorubicine, for its determination as parent molecules and its metabolites Doxorubicinol, Doxorubicinone, Doxorubicinolone, and 7-Deoxydoxorubicinone in Mouse Plasma [34,35,36]. Some liposomal systems incorporating AB induced cell cytotoxicity and apoptosis, destruction of mitochondrial membrane potential in vitro and inhibition of tumors in vivo in mice by intratumoral injection [4,18,19,20]. The present study extends our previous experience in preparing and characterizing birch extracts enriched in betulin, betulinic acid and lupeol (TT), to build PEGylated liposomes and NLC, which incorporate these molecules [1,7,21,25]. Also, the former in vitro findings confirmed their apoptotic effects on melanoma and Walker 256 cells, as compared to Doxorubicin [18,36].

Little experimental data is available regarding in vivo experiments on rats by intravenous administration using liposome and NLC nanoformulations of betulinic acid and TT extracts, and moreover, delivery to Walker 256 tumor-bearing rats by sonoporation with co-incubated microbubbles as contrast agents. That is why we tried to fill this knowledge gap, by adding value to the present experimental data.

Using UHPLC-QTOF-ESI^+^MS and multivariate statistics, the parent molecules AB, TT components, Doxo and their metabolites were identified in tumor tissues, plasma, or eliminated in urine. Meanwhile, some toxicity biomarkers (creatinine, p-cresyl sulfate and creatine, arginine and dimethylarginine, hippuric acid and indoxyl sulfate) were identified.

The working hypothesis and steps of these experiments were defined and presented above (Figure 1 and Figure 2) and the results demonstrate the different effects of liposomes vs. NLC with encapsulated AB or TT extract as compared to Doxorubicin, as free or encapsulated in liposomes and NLC.

Following a detailed characterization of sizes and encapsulation efficiency of these molecules (see Section 3.1) into nanoformulations, e.g., Doxorubicin (LDOXO, NLCDoxo), pure betulinic acid (LAB, NLCAB) and TT (LTT, NLCTT), controlled doses from each type of molecule were administered to rats, according to the protocol mentioned above.

According to the data presented in Section 3.2, no significant modifications of rat mean weight after treatment were noticed, excepting the larger SD variation in the case of tumor-bearing controls Ct and Doxo-treated groups. The tumor weights ranged from mean values of 6 to 8 g (Ct and Doxo) to 17–23 g after administration of liposomal formulations and less (11–12 g) for NLC formulations. NLCTT induced the death of all rats at the time of administration by pulmonary thromboembolism (data presented in a future paper). In general, the weight of tumors was associated with lower water consumption and the volume of urine collected after 12 h from the last administration. Complementary information will be provided by the CEUS images and histological data, to be reported later, separately in a future paper.

Here, this exploratory study focused mostly on the identification and fate of the targeted molecules, their presence as parent molecules, fragments and metabolites in plasma and tumor tissues after rats’ euthanasia, as well elimination in urine. By the UHPLC-QTOF-ESI^+^MS analysis, the parent ions, the fragments specific to each molecule and metabolites > 380 Da were identified, in parallel with pure standards, as presented in Section 3.3. These data were confirmed by other studies using pure AB and B molecules [37,38].

Using the untargeted approach and ANOVA statistics, the main metabolites of Doxorubicin were identified mainly in plasma, namely Docorubicinol (Doxomet1) and Doxorubicinone (Doxomet2), while Doxo as parent molecule and its fragment were found in the tumor tissue, especially when free Doxo and LDoxo were sonoporated. Based on UHPLC-MS peak intensities, a semi-quantitative evaluation and the distribution of Doxo, Doxomet1 and Doxomet2 in plasma (P), tumor (T) or elimination in urine (U) was achieved, as presented in Figure 5. It was shown that not only the intact Doxo molecule, but also its metabolites (Doxomet1 and Doxomet2) may play an important role in the accumulation in tumor and plasma, without significant release in urine. The accumulation of intact Doxo and its metabolites was almost equally distributed in T (T-Doxo), while Doxomet 1 was dominant in plasma (P-Doxo). Meanwhile, NLCDoxo, which induced a low survival rate, showed a lower accumulation in tumor tissue, but more in plasma, together with its metabolites (Doxomet2). When LDoxo was administered, the accumulation of Doxo and its metabolites accumulated in T and P, and was largely released in U (U-LDoxo). Administration of NLCDoxo induced accumulation in P, less in T and very low release in U, its bioavailability being lower than free or LDoxo. This finding correlated with the low survival rate of this group and the identification of metabolic toxicity biomarkers (increased creatine in tissues, creatinine and indoxyl sulfate in urine). Decreased ratios of Arg/DMA were also correlated with a stronger toxicity of free Doxo.

The same ANOVA analysis was applied to compare the levels of AB or B, AB and LU in plasma, tumor tissue and elimination in urine, when pure AB and TT nanoformulations were administered, respectively (Figure 4a–d). The PLSDA score plot, the PCA biplot, VIP scores and the heatmap showed significant discrimination among the sample profiles, being found in plasma parent molecules of AB, B and ABmet2 (originating from LAB and LTT). Meanwhile, in tumor tissue, we identified less polar molecules and fragments (LU, LUf2, Bf1, Bf2) and in urine, more polar metabolites (ABmet1, Bmet1 and Bmet2). Complementary information was offered by ANOVA post hoc graphs (Figure 4e), confirming that AB and ABmet2 levels as well as B were dominant in plasma, while in tissue only LU was identified as a parent molecule. B metabolites were mostly eliminated in urine. LU, as well as the other molecules, excepting Bf1, especially the ones delivered as NLC formulations, showed lower accumulation in tumor tissue, explained by lower availability. Based on UHPLC-MS peak intensities, a similar semi-quantitative evaluation of the distribution in P, T and U of AB, B and LU depending on the type of nanoformulation was achieved, as presented in Figure 6. The sonoporation of LAB and NLCAB showed similar behaviors, showing a higher retention of ABmet2 in plasma, and similarly, but at lower levels in tumor tissue and stronger elimination in urine. ABmet2 proved to be the retained and available molecule. When LTT was administered, components B, Bmet1 and Bmet2 showed higher retention in plasma as compared to tumor retention, where B and Bmet2 followed by ABmet1 and LU had higher accumulation. Urine elimination was more intense for B, Bmet2 and ABmet1. Also, we identified increased levels of toxicity biomarkers, e.g., increased creatine in tissues, creatinine and indoxyl sulfate in urine and decreased ratios of Arg/DMA, especially for NLCAB and LTT.

Table 6 integrates the main findings, trying to rank the different impacts of the nanoformulations, the specific distribution of parent molecules and their metabolites, with implications on their antitumor effects in vivo.

Considering the survival scores and the tumor weights, as compared to free Doxo, L Doxo (having scores up to 2), all the other formulations (NLC Doxo, LAB, NLCAB and LTT) showed higher scores (up to 8), suggesting a stronger tumor inhibition. Regarding the distribution, free Doxo was found in tumors, while its metabolites were found in plasma and tumors, without significant elimination in urine. The nanoformulations including AB or TT components showed a more intense persistence in plasma of parent molecules and met2 derivatives, while met1 was mostly eliminated in urine. The LU component of TT was proved to be accumulated in the tumor tissue, being preferred as a nonpolar component.

**Toxicity biomarkers.** Initially, by ANOVA untargeted approach, we identified and compared the profiles of eight putative biomarkers of toxicity either in plasma, tumor tissue or urine, as shown in Figure 7. The PCA biplot and VIP scores > 1 showed significantly increased distribution of creatinine, p-cresyl sulfate in plasma versus creatine, Arg, DMA, Hippuric acid and Indoxyl sulfate in tumor tissues. The impact of these data is related to hepatic or renal stress, reflected by these biomarkers. Hippuric acid, a metabolite of glycine, is a potential biomarker of cancer, increased in tumor tissues, and Indoxyl sulfate is a uremic toxin derived from dietary proteins metabolized by gut bacteria and found in urine. It is normally excreted in urine, but elevated levels in the blood can indicate chronic kidney disease due to reduced renal clearance. Creatine’s role in tumor tissues is complex, with both tumor-suppressing and tumor-promoting effects (as an energy source for cell growth, invasion, and metastasis). The Arg/DMA ratio is an indicator of the activity of nitric oxide synthase (NOS) since in tissues, L-arginine serves as a substrate for producing nitric oxide (NO), a key molecule related to endothelial dysfunction and cardiovascular health, crucial for various cellular functions like signaling and vasodilation. A low arginine/DMA ratio in blood and tissues is linked to increased cardiovascular risk, since DMA is an endogenous inhibitor of NOS.

The heatmap confirms these findings and moreover identifies Doxo, L-Doxo and NLC-Doxo as formulations which increased the levels of creatinine, p-cresyl sulfate and uric acid in plasma. In tumor tissue, the above-mentioned molecules had increased levels when L-Doxo and NLC-AB were administered.

Considering the UHPLC-MS peak intensities, as an indicator of semi-quantitative evaluation, as compared to Ct, the plasma creatinine levels were not significantly different, while p-CS was significantly increased in groups Doxo, LDoxo and NLCDoxo (Figure 8a). In tumor tissues, creatine levels were increased comparatively to Ct, in groups Doxo, LDoxo and NLCAB. These data suggest that Doxo and LDoxo, as well NLC formulations of Doxo and AB were more toxic. Significant increase in urine creatinine was also observed for group Doxo and NLCAB. The ratio of Arg/DMA was significantly decreased for Ct, NLCAB, LTT in plasma, while in tumor tissue this ratio did not show significant variations as compared to Ct (except for NLC Doxo, with a significant decrease).

## 5. Conclusions

This exploratory study focused on the metabolomic approach for identifying betulins’ and their metabolites responsible for the Walker 256 tumor inhibition in Wistar rats, using UHPLC-QTOF-ESI^+^MS analysis of plasma, tumor tissue and urine, assisted by Metaboanalyst 6.0 statistics. Their entrapment in PEGylated liposomes and NLC-nanolipid carriers, compared to Doxorubicin, delivered by intravenous administration and microbubble-assisted sonoporation showed distinct effects.

The processed data adds new knowledge regarding the metabolization of betulins in vivo, the roles and bioactivity of their main metabolites and their fate as compared to Doxorubicin, free or in similar nanoformulations. Liposomal formulations proved to be, in general, more bioavailable, with less toxicity than NLC formulations. NLC formulations, especially the ones including TT extract with less polar components, proved to be more toxic despite its lower bioavailability.

We are aware of the limitations of this study; despite the good control of nanoformulation size and encapsulation efficiency of molecules, the co-incubation of nanoformulations with ready-made microbubbles, non-chemically conjugated, was difficult to control and their synergistic effects were difficult to follow. The CEUS imaging and histological data collected from these investigations will complement the information and may draw supplementary conclusions about the diverse metabolic and morphological effects of these formulations upon Walker 256 tumor bearing rats.

Future research will aim to identify the dose–effect relationships using i.v. administration for an improved antitumor activity of these nanoformulations. Meanwhile, elucidation of the metabolic pathways involved and the appropriate ratios between cytotoxicity and benefits of betulins in experimental animals will be followed.

## Figures and Tables

**Table 3:** Specific parent ions [M+1] and main fragments of pure standards used in this study. The retention time (Rt) is also included, according to the analytical protocol (see Materials and Methods).

Pure Standard	Rt (min)	Parent Ion [M+1]	Main Fragments (>380 Da)
Doxorubicin (Doxo)	4.3	544.1360	397.0619 (Doxof1)
Betulin (B)	8.3	443.3068	425.3474 (Bf1); 393.2769 (Bf2)
Betulinic acid (AB)	9.9	457.6238	439.2293(ABf1); 429.2957 (ABf2)
Lupeol (LU)	10.6	427.2731	409.2744(LUf1); 383. 2850 (LUf2)

**Table 4:** Parent molecules, fragments and metabolites detected by UHPLC-QTOF-ESI^+^MS in plasma, urine or tumor tissues: Retention times (Rt), abbreviations and details about their structures. Metabolites are presented in italic font.

[M+1]	Rt (min)	Abbreviation	Parent Ions, Fragments and Metabolites (Structures)
383.2850	10.2	LUf2	LU fragment (loss of CH_3_ and C_2_H_4_)
*385.2717*	*8.3*	*Bmet1*	*Metabolite of B (loss O and conversion of O-ethyl ketone to acid)*
393.2769	8.2	Bf2	B fragment (decarboxylation)
*395.2224*	*8.1*	*Bmet2*	*Metabolite of B (loss O and O+demethylation and desaturation)*
409.2744	10.5	LUf1	LU fragment (loss of CH_3_)
425.3474	8.3	Bf1	B fragment (loss of OH)
427.2731	10.6	LU	LU parent
429.2957	9.9	ABf2	AB fragment (loss of CO)
439.2293	9.9	ABf1	AB fragment (loss O and desaturation)
443.3068	8.3	B	B parent
*445.2791*	*9.7*	*ABmet1*	*Metabolite of AB (loss of CH_2_ and hydroxylation)*
457.6374	9.9	AB	AB parent
*459.2930*	*8*	*Bmet3*	*Metabolite of B (oxidation of ethyl to carboxylic acid)*
*487.3289*	*9.6*	*ABmet2*	*Metabolite of AB (conversion of CH_2_ to HCOOH)*
544.1363	4.3	Doxo	Doxo parent
*545.3606*	*2.7*	*Doxomet1*	*Metabolite of Doxo (Doxorubicinol)*
397.0619	4.3	Doxof1	Doxo, main fragment
*415.0691*	*2.7*	*Doxomet2*	*Metabolite of Doxo (Doxorubicinone)*

**Table 5:** Retention time (Rt), [M+1] values and HMDB ID of molecules identified in plasma, urine or tumor tissue samples, as putative biomarkers of toxicity after administration in vivo of all sample variants (see Table 2).

Rt (min)	Molecule	[M+1]	HMDB ID
1.6	Arginine (Arg)	175.1057	HMDB0000517
1.8	Dimethylarginine (DMA)	203.0370	HMDB0251395
2.3	Creatine	132.0923	HMDB0000064
2.5	Creatinine	114.0560	HMDB0000562
5.2	Uric acid	169.1079	HMDB0000289
7.9	Hippuric acid (Hp)	180.1242	HMDB0000714
8.0	Indoxyl sulfate (IS)	214.2369	HMDB0000682
8.9	p-Cresyl sulfate (pCS)	189.1486	HMDB0011635

**Table 6:** Ranking of survival scores, tumor weight and relative distribution of parent molecules (Doxo, AB, B, LU) encapsulated in nanoformulations (LDoxo, NLCDoxo, LAB, NLCAB, LTT) and their metabolites in tumor tissue, plasma and elimination in urine, after their i.v. administration by microbubble-assisted sonoporation. * 5—lowest survival score; ** 6 lowest tumor weight.

	Survival Score *	Tumor Weight **	Doxo	Doxo met1	Doxo met2				
Doxo	1	1	T > U > P	P > T > U	P > T > U				
LDoxo	1	2	P~T~U	T > P~U	P~U > T				
NLCDoxo	5	3	P > T~U	P > T~U	P > T > U				
			AB	AB met1	AB met2	B	B met1	B met2	LU
LAB	2	6	P > U > T	U > P~T	P > U > T				
NLCAB	4	4	P > U > T	U > P > T	P > U > T				
LTT	3	5	P~T > U	U > T > P	P > U > T	P > U > T	P > T > U	U > P > T	T > U > P

## Data Availability

The data presented in this study are available on request from the corresponding author due to ethical restrictions.

## Supplementary Materials

The following supporting information can be downloaded at: https://www.mdpi.com/article/10.3390/metabo15110723/s1, Supplementary File S1—Extracted Ion Chromatograms and MS data for AB, TT and Doxo in plasma, tissue and urine after i.v. administration of PEGylated liposome and NLC formulations.

## Abbreviations

The following abbreviations are used in this manuscript:

AB	Betulinic Acid
B	Betulin
DLS	Diffraction Light Scattering
Doxo	Doxorubicin
MB	Microbubbles
MPBE	Methanol:Isopropanol:Methyl *t*-butyl Ether (2:1:1)
MS	Mass Spectrometry
NLCs	Lipid Nanostructured Carriers
P	Plasma
PBS	Saline Phosphate Buffer
PDI	Poly Dispersibility Index
PEG	Polyethylene Glycol
PLGA	Poly (lactic-co-glycolic acid
SALD	Laser Diffraction
T	Tumor Tissue
TT	Pentacyclic Triterpenoids
U	Urine
UHPLC	Ultra-High Performance Liquid Chromatography
