# Supplementary material for: Delivery and Metabolic Fate of Doxorubicin and Betulin Nanoformulations In Vivo: A Metabolomics Approach
Source: Metabolites. 2025 Nov 5;15(11):723. doi: 10.3390/metabo15110723 (PMC12654472; doi:10.3390/metabo15110723)
Supplement: Supplementary file 1 [file metabolites-15-00723-s001.zip › metabolites-3938370-supplementary.pdf]

Supplementary file S1. Figures 1-3. Extracted Ion Chromatograms and MS data for AB, TT and Doxo in plasma, tissue and urine after i.v. administration of PEGylated liposome and NLC formulations.

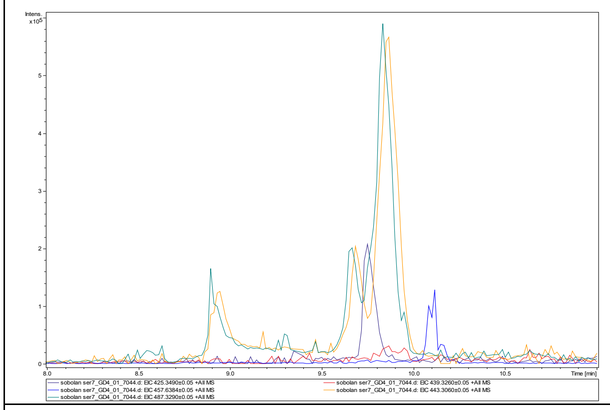

(a) m/z= 457.6384 (blue); 425.3474 (grey); 487.3299 (green) and 443.3068 (orange);

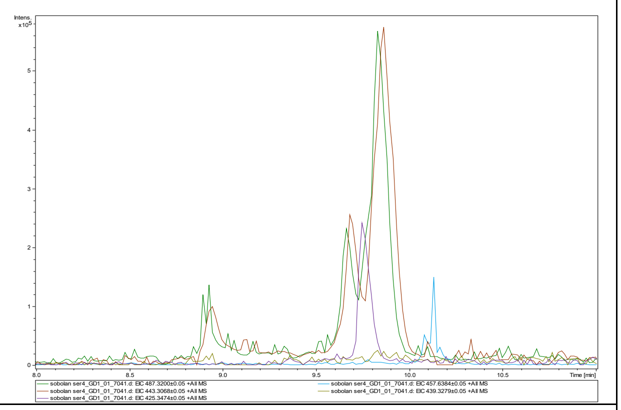

(b) m/z= 457.6384 (light blue); 425.3474 (violet); 487.3299 (green) and 443.3068 (orange);

Figure 1A. EIC for AB metabolites in Plasma after L-AB (a) and NLC-AB (b) i.v. administration. RT= 9.7-10.1 min

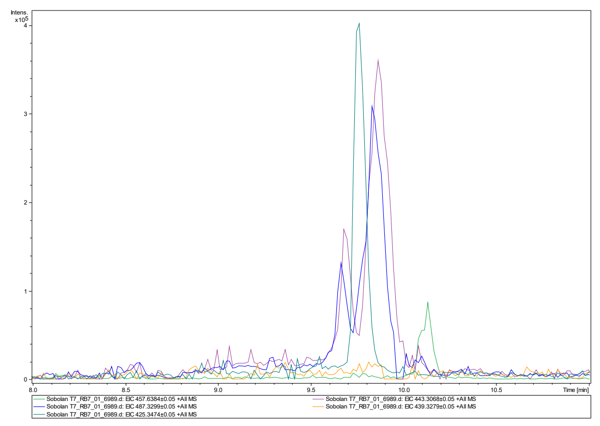

(a) m/z= 457.6384 (green); 425.3474 (deep green); 487.3299 (blue) and 443.3068 (violet)

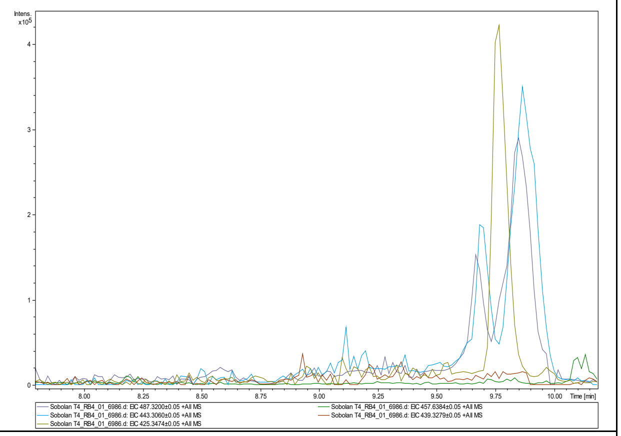

(b) m/z= 457.6384 (green); 425.3474 (grey); 487.3299 (violet) and 443.3068 (light blue)

Figure 1B. EIC for AB metabolites in Tumor tissue L-AB (a) and NLC-AB (b) after i.v. administration.; at RT= 9.7-10.1 min

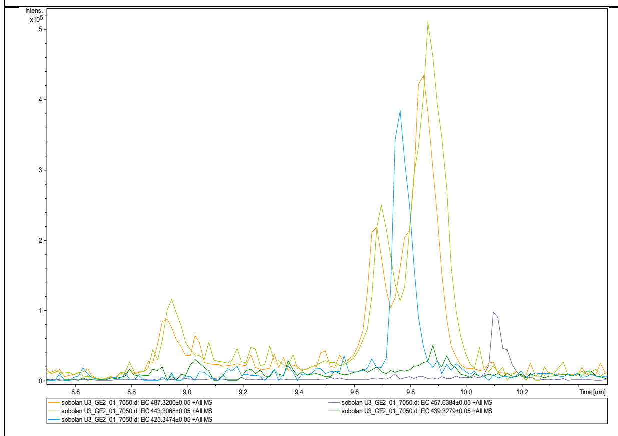

(a) m/z= 457.6384 (blue); 425.3474 (light blue); 487.3299 (orange) and 443.3068 (green)

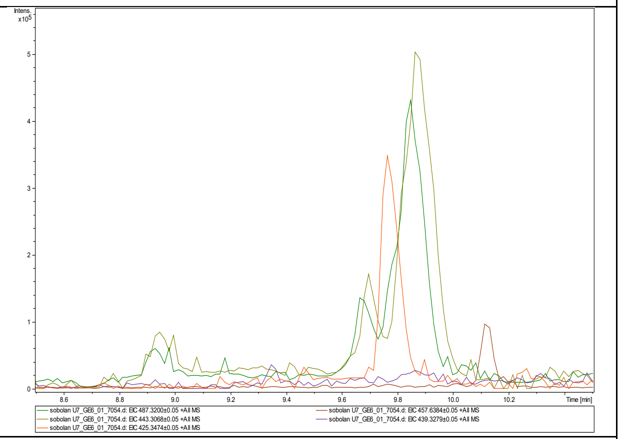

(b) m/z= 457.6384 (violet); 425.3474 (red); 487.3299 (deep green) and 443.3068 (gray)

Figure 1C. EIC for AB metabolites in Urine L-AB (a) NLC-AB (b) after i.v. administration. Main peaks at m/z= 457.6384 (**1**); 425.3474 (**2**); 487.3299 (**3**) and 443.3068 (4); at RT= 9.7-10.1 min

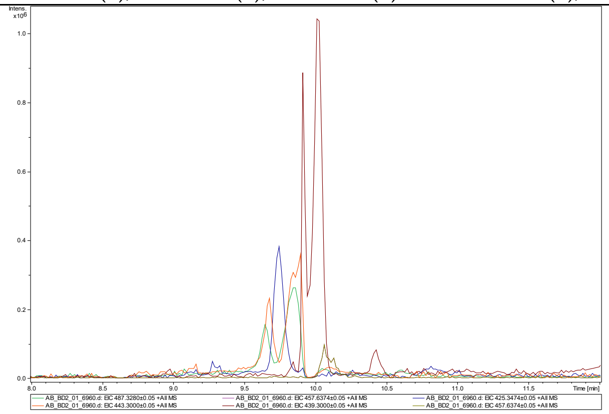

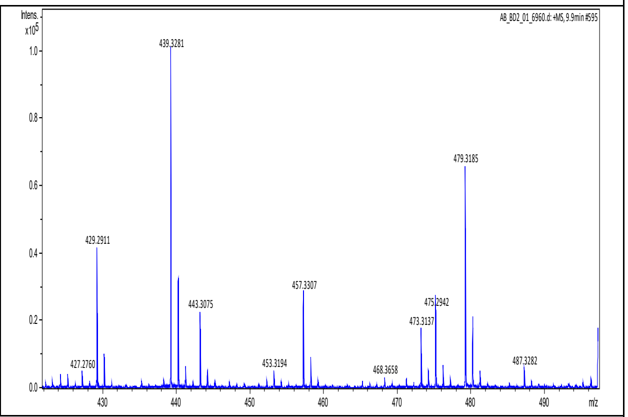

Figure 1D. Left: EIC for AB pure standard: m/z= 457.6384 (deep green); 479.3125 (green) 443.3068 (red) and 439.3260 (brown). Right: Main ion fragments identified in BA pure standard

Figure 1 A-C. EIC graphs of AB metabolites in plasma, tissue and urine after i.v. administration pf nanoformulations. **Figure 1D.** EIC for AB standard (a) and its MS spectra (b). Main peaks at RT= 9.7-10.1 min

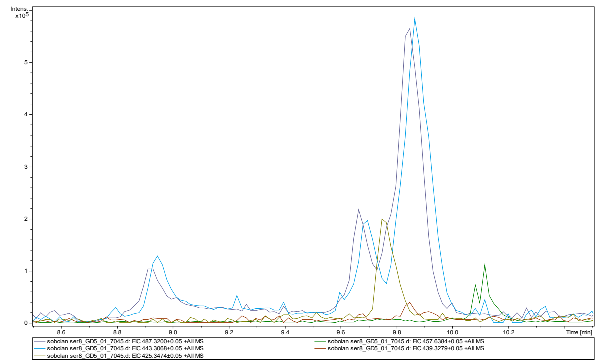

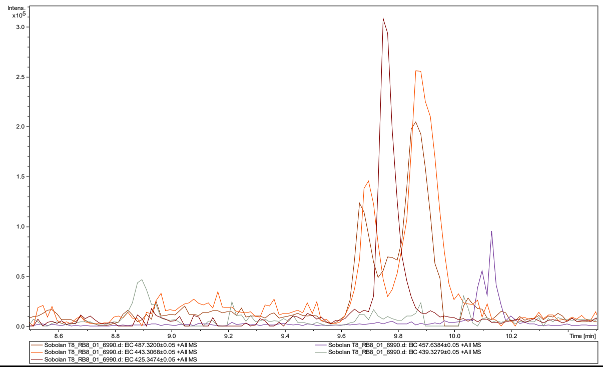

Figure 2A. a. EIC for TT metabolites in Plasma after L-TT treatments. at m/z= 457.6384 (green); 425.3474 (deep green); 487.3299 (grey) 443.3068 (light blue); 439.3260 (deep red)

Figure 2B. EIC for TT metabolites in tumor tissue after L-TT treatments. m/z= 457.6384 (violet); 425.3474 (brown); 487.3299 (grey) 443.3068 (red); 439.3260 (deep green)

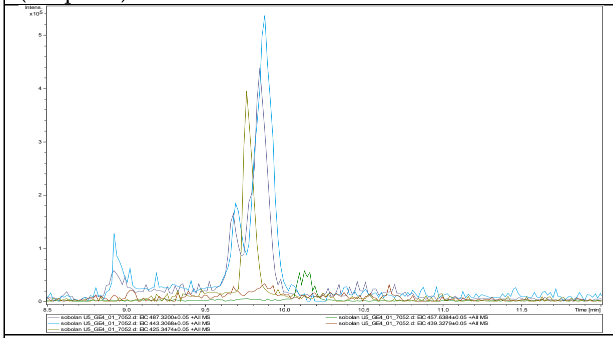

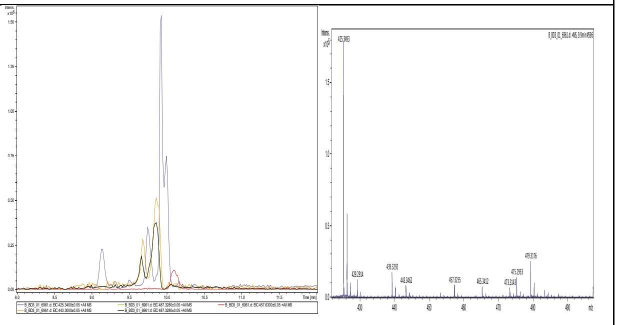

Figure 2C. c. EIC for TT metabolites in urine after L-TT treatments. m/z= 457.6384 (green); 425.3474 (deep green); 443.3068(light blue); 439.3260 (deep red)

Figure 2D. EIC for B (a) and its MS spectra. 425.3474 (blue); 443.3068(orange); 393.2769 (deep red)

Figure 2A-C. EIC graphs of metabolites after L-TT i.v. administration. **Figure 2D.** EIC for B pure standard EIC (left) and fragments in MSn spectra (right)

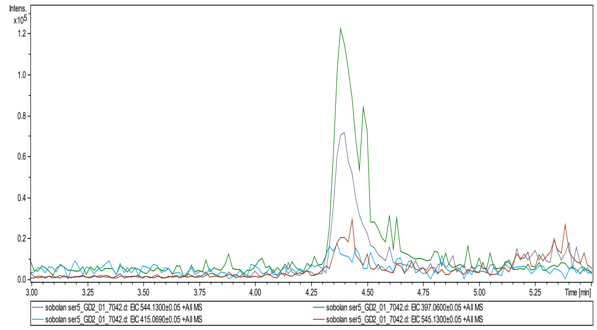

a. EIC for Doxo metabolites in Plasma after L-Doxo i.v. administration: m/z= 544.136 (grey); 545.136(deep red); 415.069 (light blue) and 397.061 (green). Rt= 4.3 min

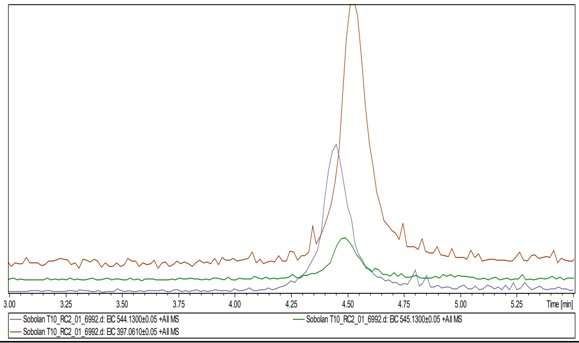

b. EIC for Doxo metabolites in tumour tissue after L-Doxo i.v. administration: m/z= 544.136 (grey); 545.136(green) and 397.061 (deep red). Rt= 4.5 min

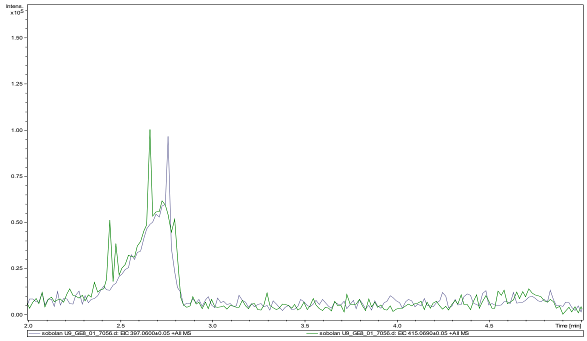

c. EIC for Doxo metabolites in urine after L-Doxo i.v. administration: m/z= 415.069 (green) and 397.061 (blue). Rt=2.3-2.6 min

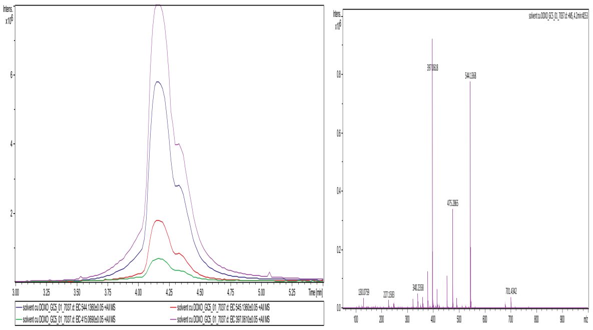

d. EIC for free Doxo (left) and its MS spectra (right). Main peaks at m/z= 544.136 (blue); 545.136(red); 415.069 (green) and 397.061 (violet) at Rt= 4.2 min

Figure 3. (a-c) EIC graphs of metabolites after L-Doxo i.v. administration. Main peaks identified at at RT= 4.2, 10.2, 10.2, and 2.1 min, respectively. (d)EIC graph for free Doxo (pure standard) and MSn
